# Emergency Medicine Residents’ Performance with National Institutes of Health Stroke Scale and Its Impact on Key Stroke-care Metrics

**DOI:** 10.5811/westjem.39671

**Published:** 2025-10-21

**Authors:** Matthew Roces, Trinidad Alacala-Arcos, Newton Addo, Michael Boyle, Meghan Hewlett, Reginald Nguyen, Angela Wong, Christopher R. Peabody, Debbie Y. Madhok

**Affiliations:** * University of California Los Angeles, Department of Emergency Medicine, Los Angeles, California; † University of California Irvine, School of Medicine, Irvine, California; ‡ University of California San Francisco, Department of Emergency Medicine, San Francisco, California

## Abstract

**Background:**

Emergency medicine (EM) physicians commonly use the National Institutes of Health Stroke Scale (NIHSS) to assess acute ischemic strokes in community settings. However, this assessment is often led by neurology residents in academic teaching hospitals. We implemented a quality improvement intervention to improve EM resident comfort with the NIHSS and to assess if EM resident-led NIHSS evaluation prolonged key stroke metrics, such as door-to-CT (DTCT), door-to-needle (DTN), or door-to-groin puncture (DTGP) times, which may affect stroke outcomes.

**Methods:**

This prospective observational comparison analyzed all patients with acute ischemic strokes at the Zuckerberg San Francisco General Hospital, a Level I trauma center from April 2021–October 2022. We implemented the intervention from April 2022 –October 2022 which included NIHSS certification for all residents and attendings. Both EM and neurology residents recorded NIHSS scores separately for each patient and scores were revealed to each resident during patient care once completed. We then compared stroke metrics between pre- and post-intervention periods.

**Results:**

There were 247 and 122 strokes included in our analysis, pre- and post-intervention, respectively. Overall, 58% (n=213) of all patients were female, 33% were Asian (n=123), and Cantonese was the second most common language after English (15%, n=54). Mean overall NIHSS scores were similar between EM and neurology residents, 6.6 (IQR = 2, 10) and 6.7 (IQR = 1, 10), (p < 0.001), respectively, with substantial agreement between groups (84.4%, κ = 0.63). Median DTCT times were 25 and 28 minutes (p=0.2), DTN times were 38 and 35 minutes (p=0.7), and DTGP times were 94 and 110 minutes (p=0.1) for pre- and post-intervention groups, respectively.

**Conclusion:**

The NIHSS is one element of stroke evaluation and management that can impact stroke metrics. Our intervention found that EM resident-led NIHSS assessment did not prolong DTCT, DTN, and DTGP times and met nationally established goals.

## INTRODUCTION

Stroke remains one of the leading causes of death and disability in the United States, affecting nearly 800,000 people annually, 87% of which are ischemic. 1 The National Institutes of Health Stroke Scale (NIHSS) is a 15-item neurologic assessment used in the evaluation of acute ischemic strokes 2 and is strongly predictive of outcome after stroke. 3–5 This survey is used to assess severity, with higher scores correlating with more severe strokes evidenced by more profound neurologic deficits. 6 The NIHSS score aids in treatment decisions, either with intravenous tissue plasminogen activator (tPA) or endovascular thrombectomy (EVT). Key metrics in stroke management include time from hospital presentation to computed tomography (CT) imaging (door-to-CT time), thrombolytic therapy (door-to-needle time), and EVT (door-to-groin puncture time), as improvement in these measures correlates with decreased in-hospital mortality, improved clinical outcomes, and reduced one-year mortality. 7–9 The literature is sparse on NIHSS training among emergency medicine residents as it relates to the management and outcomes of acute ischemic strokes. The American Heart Association (AHA) updated its Stroke Phase III guidelines to establish national goals of door-to-needle times <60 minutes and door-to-groin puncture times <90 minutes for patients arriving directly to a stroke center. 10

While the NIHSS is used in community emergency departments (ED) across the US, it is not uncommon for the NIHSS is to be conducted by neurology residents at academic hospitals. This poses a potential area of improvement in emergency medicine (EM) training, especially for graduates who plan to practice in community settings. We sought to determine whether a stroke quality improvement (QI) intervention could improve EM residents’ comfort with the NIHSS and stroke management. (We analyzed these surveys of resident comfort levels and will discuss them in a separate paper.) We also aimed to see whether this intervention would influence acute stroke metrics, including door-to-CT, door-to-needle, and door-to-groin puncture times before and after our intervention.

## METHODS

Our group sought oversight from our local Institutional Review Board (IRB) who determined our project to be a quality improvement initiative and not research. We analyzed all data with R Version 4.1 (R Foundation, Vienna, Austria), using Chi-square or Fisher’s exact tests for categorical data and using Wilcoxon rank-sum tests for continuous data.

This prospective observational comparison included all patients with ischemic strokes presenting to the Emergency Department at Zuckerberg San Francisco General Hospital, a public safety net hospital and Level I trauma center, from April 2021–October 2022. We implemented our intervention in April 2022 and included NIHSS certification for all EM residents in their second through fourth post-graduate years (PGY 2–4) and attending physicians. This accreditation process involved a 6-part 8 hour online module through the American Heart Association11. During this six-month pilot, EM and neurology residents separately collected and calculated the NIHSS via a REDCap survey form which can be viewed in the supplementary section. NIHSS was used as the main clinical decision making tool for stroke management. Other tools, such as the modified Rankin scale, were not used. The examination itself was performed in tandem by both the neurology and EM residents. The survey results themselves were blinded to each other until both scores were submitted. The results subsequently displayed a side-by-side comparison of NIHSS scoring by both EM and neurology teams allowing for discussion of similarities and differences in scoring. Both EM and neurology were involved in scoring and deciding if the patient would be imaged in the post intervention group. The mean overall NIHSS scores of approximately 30 matched surveys were similar between the two groups which was discussed in a separate paper12. There was no designated time for score revelation, however, most would happen during patient care (e.g. while patient was in CT scan).

**Population Health Research Capsule**

What do we already know about this issue?

*Data are limited comparing key stroke metrics between emergency medicine and neurology residents.*

What was the research question?

*Do NIHSS-trained EM residents prolong key stroke metrics compared to neurology residents?*

What was the major finding of the study?

*There was no significant difference in door-to-CT, door-to-needle, or door-to-groin puncture times.*

How does this improve population health?

*Establishing NIHSS training for EM residency programs allows for more competent evaluation and management of ischemic strokes.*

As primary outcomes, we compared DTCT, DTN, and DTGP times for all ischemic strokes occurring before and after the intervention periods. We excluded 6 patients with DTCT times greater than 8 hours and 2 patients with DTN and DTGP times greater than 6 hours from the analysis from all ischemic strokes. There were no other exclusion criteria. Our study had 80% power at a two-tailed alpha to detect absolute differences in DTCT times less than 30 minutes greater than or equal to 15%.

## RESULTS

**Figure 1:**
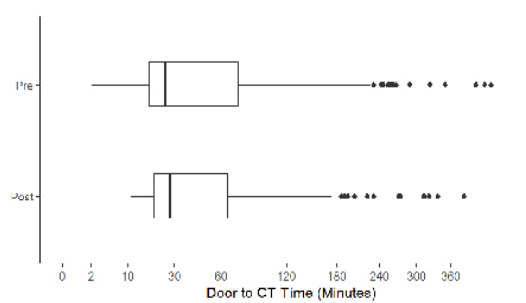
Door-to-computed tomography times in minutes in a study of National Institutes of Health Stroke Scale performance by emergency medicine residents compared with neurology residents.

**Figure 2:**
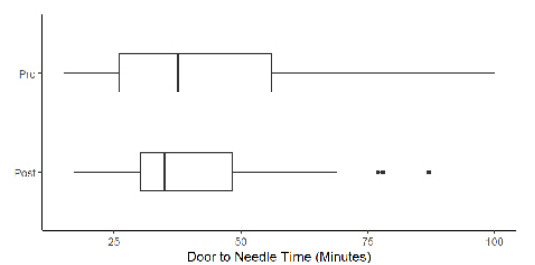
Door-to-needle time in minutes in a study of National Institutes of Health Stroke Scale performance by emergency medicine residents compared with neurology residents.

**Figure 3:**
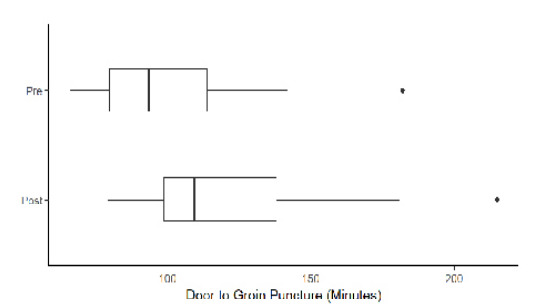
Door-to-groin puncture time in minutes in a study of National Institutes of Health Stroke Scale performance by emergency medicine residents compared with neurology residents.

**Figure 4:**
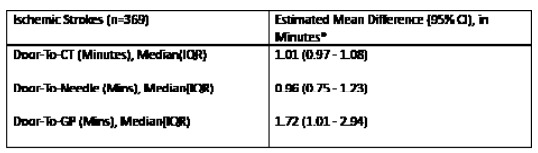
Estimated mean difference in minutes in a study of National Institutes of Health Stroke Scale performance by emergency medicine residents compared with neurology residents. *CI*, confidence interval; *CT*, computed tomography; *IQR*, interquartile range.

**Figure 5:**
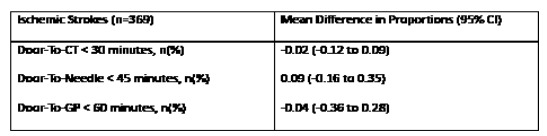
Estimated mean difference in proportions (95% CI) in a study of National Institutes of Health Stroke Scale performance by emergency medicine residents compared with neurology residents. *CI*, confidence interval; *CT*, computerized tomography.

Our analysis included 369 ischemic stroke patients presenting to the ED, with 247 (67%) strokes analyzed prior to intervention and 122 (33%) analyzed after intervention. 9.76% of all patients in our analysis had groin puncture (n=36). The table describes baseline demographics and characteristics of patients with ischemic strokes. Overall, 213 patients (58%) were female, and 123 (33%) were Asian, with Cantonese the second most common language spoken after English (54, 15%). There was no statistically significant difference between the pre- and post-intervention group demographics and characteristics with regard to sex, race, and language. Median door-to-CT times were 25 minutes (interquartile range [IQR] 8–74) during the pre-intervention period and 28 minutes (IQR 20–65) for the post-intervention period (*P*=.2). Median door-to-needle times were 38 minutes (IQR 26–56) and 35 minutes (IQR 30–48) pre- and post-intervention, respectively (*P*=.7). A total of 122 patients (54%) in the post-intervention period and 56 (53%) in the pre-intervention had door-to-CT <30 minutes (*P*=.8), while 37 patients (80%)in the pre-intervention and 17 (77%) in the post-intervention period had door-to-needle times <60 minutes (*P*=.8). Median door-to-groin puncture times were 94 minutes (IQR 80–114) and 110 minutes (IQR 99–138) pre- and post-intervention, respectively (*P*=.1). There was no statistically significant difference in acute stroke metrics between the pre- and post-intervention. Figures 1–5 provide a comparison of door-to-CT, door-to-needle, and door-to-groin puncture times pre- and post-intervention.

## DISCUSSION

Efficient and timely evaluation and management of acute ischemic strokes are imperative in improving morbidity after stroke. The NIHSS is one part of stroke management that can impact key stroke metrics, such as door-to-CT, door-to-needle, and door-to-groin puncture times. As the NIHSS is at the crux of management of acute stroke, particularly in community EDs, our goal was to assess whether emergency physicians trained in using the NIHSS and performing the NIHSS alongside neurology residents at an academic institution would affect key stroke metrics. We found that EM resident-led NIHSS assessment had no impact on door-to-CT, door-to-needle, and door-to-groin puncture times. During both the pre-intervention and post-intervention periods, most door-to-needle times were <60 minutes, meeting the national goal of the AHA Stroke Phase III guidelines. Most of the median door-to-groin puncture times, however, were >90 minutes for both the pre- and post-intervention periods, which exceeded the door-to-device goal set by the AHA. 9 We did not investigate the reasons for longer door-to-groin puncture times; the impact of the NIHSS on these times specifically remains unknown.

Our QI initiative demonstrated that it is possible to incorporate NIHSS training within EM residency training without having the detrimental effect of prolonging stroke metrics. While there is limited literature on NIHSS training among EM residency programs, one study did survey neurology residents’ comfort with assessing and managing acute ischemic strokes with components including NIHSS evaluation and tPA. This 10-year survey showed significantly increased reported proficiency and comfort levels. 12 However, more studies are needed to further investigate this relationship.

## LIMITATIONS

There were several limitations to our study. While the NIHSS scores were blinded, the exam itself was done in tandem. While residents were instructed not to disclose scoring during the examination, the exam itself was not completely blinded. The NIHSS surveys were voluntarily submitted by EM and neurology residents. Thus, not every stroke-activated patient had documented surveys. Of 122 post-intervention strokes there were 29 surveys completed by both EM and neurology residents. Additionally, surveys were anonymized, and the level of training for each resident was not recorded. This could have had some unmeasured effect on door-to-CT, door-to-needle, and door-to-groin puncture times, wherein more senior residents could have made their assessments and management decisions faster than junior residents.

Further, because the dates of the surveys collected were not recorded, we did not perform a time series analysis, which could have helped determine whether other factors led to effects on stroke metrics. A final limitation to our study was our designation of neurology residents as the gold standard for acute ischemic stroke care. Our pre-intervention metrics were all based on neurology-led NIHSS assessments; however, the variability in training and accuracy may have had an unmeasured effect. Our study was also conducted at an urban, Level I trauma center and public, safety-net hospital with 24-hour neurology consultation, which may limit generalizability. However, our study population did include a diversity of socioeconomic statuses and primary languages spoken, which could be generalized to other study populations.

## CONCLUSION

The National Institutes of Health Stroke Scale is a vital part of acute ischemic stroke assessment and can be incorporated into EM resident training without delaying critical imaging or treatment metrics. Future studies that address the limitations are necessary to further characterize EM resident impact on key stroke metrics in a variety of settings including demographics, time frame, geography, and in-house resources.

## Supplementary Information



## Figures and Tables

**Table:** Emergency department stroke metrics in a study of National Institutes of Health Stroke Scale performance by emergency medicine residents compared with neurology residents.

		Overall N=369	Pre n=247	Post n=122	*P*-value
Age	18–44	24 (6.5%)	17 (6.9%)	7 (5.7%)	.7
	45–54	29 (7.9%)	16 (6.5%)	13 (11%)	
	55–64	82 (%)	52 (21%)	30 (25%)	
	65–74	95 (%)	65 (26%)	30 (25%)	
	75–84	72 (%)	51 (21%)	21 (17%)	
	85+	67 (%)	46 (19%)	21 (17%)	
Sex	Female	156 (42%)	106 (43%)	50 (41%)	.7
	Male	213 (58%)	141 57%)	72 (59%)	
Race	Asian	123 (33%)	90 (36%)	33 (27%)	.2
	Black	77 (21%)	46 (19%)	31 (25%)	
	White	76 (21%)	48 (19%)	28 (23%)	
	Other	93 (25%)	63 (26%)	30 (25%)	
Language	Mandarin/Cantonese	54 (15%)	41 (17%)	13 (11%)	.5
	English	217 (59%)	142 (57%)	75 (61%)	
	Spanish	53 (14%)	34 (14%)	19 (16%)	
	Other	45 (12%)	30 (12%)	15 (12%)	
