# Supplementary figures and images for: Emergency Medicine Residents’ Performance with National Institutes of Health Stroke Scale and Its Impact on Key Stroke-care Metrics
Source: West J Emerg Med. 2025 Oct 21;26(6):1764–8. doi: 10.5811/westjem.39671 (PMC12698169; doi:10.5811/westjem.39671)

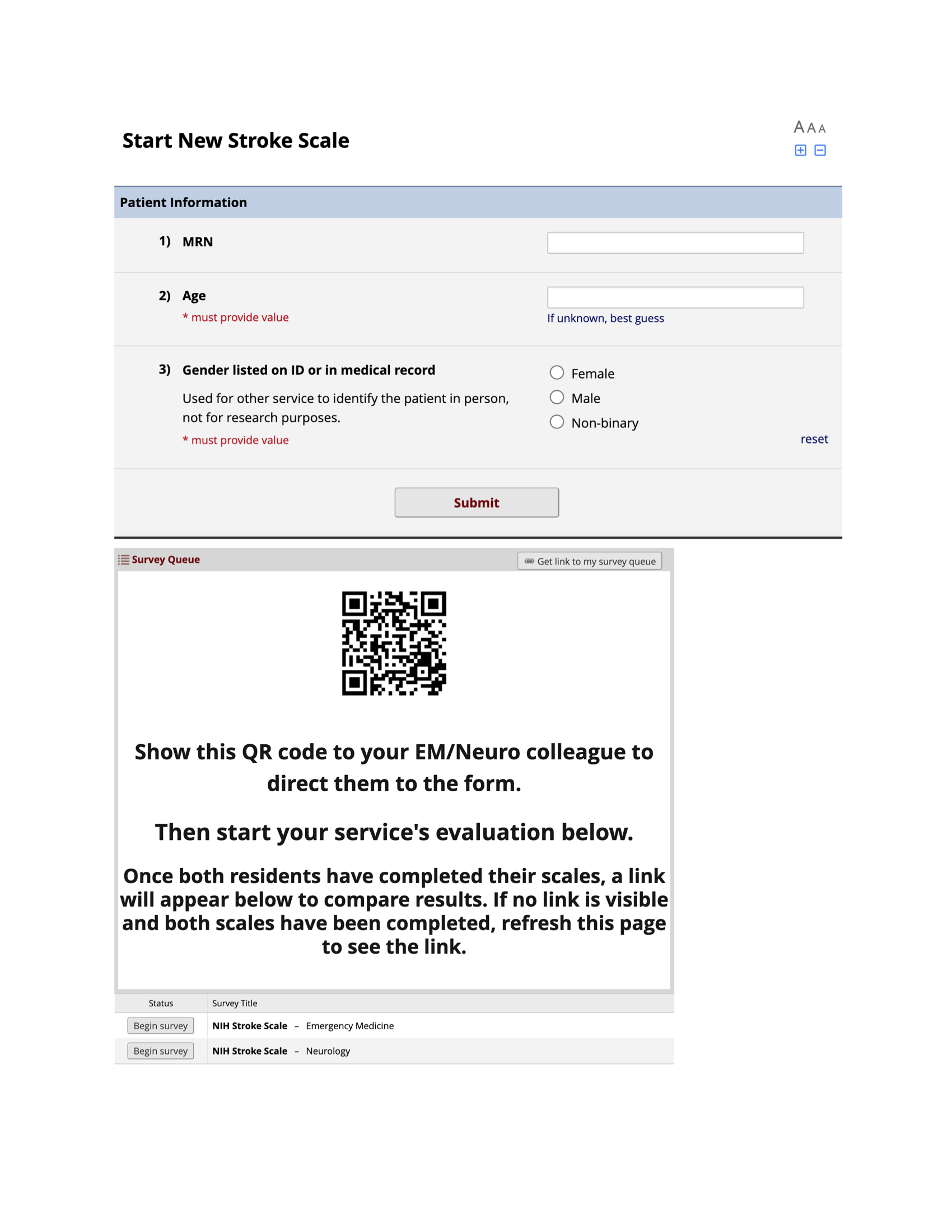

Supplement: Supplementary file 1 [file wjem-26-1764-s001.png]
